# Supplement use is common in Dog Aging Project participants, especially among dogs with orthopedic conditions, and varies by life stage

**DOI:** 10.2460/ajvr.25.06.0217

**Published:** 2025-11-14

**Authors:** Janice S. O’Brien, Dog Aging Project Consortium, M. Katherine Tolbert, Audrey Ruple

**Affiliations:** 1 Population Health Sciences Department, Virginia-Maryland College of Veterinary Medicine, Virginia Tech, Blacksburg, VA; 2 Department of Small Animal Clinical Sciences, College of Veterinary Medicine and Biomedical Sciences, Texas A&M University, College Station, TX; 3 Department of Small Animal Clinical Sciences, Gastrointestinal Laboratory, College of Veterinary Medicine and Biomedical Sciences, Texas A&M University, College Station, TX

**Keywords:** nutrition, nutritional supplements, dog nutrition, Dog Aging Project, dietary supplements

## Abstract

**Objective:**

To analyze the use of supplements reported by owners of dogs enrolled in the Dog Aging Project (DAP) to examine (1) the types of supplements being given to dogs within the DAP cohort and (2) the extent to which dogs diagnosed with specific conditions are being given supplements that are thought to improve those conditions.

**Methods:**

Supplement use, demographic variables, and owner-reported health conditions of interest were extracted from DAP survey responses collected from January 1, 2020, through December 31, 2022, from dog owners. Prevalence estimates were calculated in this cross-sectional design using survey responses from owner survey at enrollment. Free-text analysis of “other” responses was completed using Atlas.TI software to categorize additional responses.

**Results:**

Half of the dogs in this study (20,993 of 40,367 [52%]) received supplements. Of those receiving supplements, the most common supplements administered were omega-3 fatty acids (11,934 [57%]) and joint supplements (11,810 [56%]). Dog demographic characteristics were more strongly associated with differences in supplement use than owner demographic characteristics.

**Conclusions:**

Supplement use is common among dogs in the DAP cohort, with approximately half of owners reporting supplement administration. Given the frequency of their administration, veterinary professionals should discuss supplement use with dog owners.

**Clinical Relevance:**

Veterinary team professionals should consider spending more time discussing supplement use and efficacy with dog owners, especially for owners of senior pets.

Dietary supplements for humans are defined by the US FDA in the Dietary Supplement Health and Education Act of 1994 as being “a product intended for ingestion that, among other requirements, contains a ‘dietary ingredient’ intended to supplement the diet …. specifically, they must be swallowed – so, for example, topical or inhaled products are not supplements.”^1^ However, supplements intended for animals are specifically excluded from the Dietary Supplement Health and Education Act.^2^ The National Animal Supplement Council provides a voluntary labeling strategy, but this labeling method is not formally approved by the FDA. As a result, supplement labels have a wide range of claims and marketing strategies. Pet supplements are a diverse category of products. Some are multivitamins that are formulated for pets, some are dispensed by veterinarians as part of a treatment plan for various conditions (eg, urine acidifier/alkalinizer for pets with particular urinary stones), and others are not recommended for use in pets by veterinarians but continue to be sold (eg, granulated garlic for flea prevention). If supplements make claims about affecting health, they are considered drugs rather than supplements and are in the regulatory purview of the FDA.^3^

There are many studies investigating individual supplement effects on specific health conditions in animals, revealing a complex landscape of potential benefits and limitations. In several studies,^4–7^ dietary fiber supplementation has demonstrated promising outcomes for several chronic gastrointestinal conditions, which has prompted their addition to many therapeutic and over-the-counter gastrointestinal diets. Omega-3 fatty acid supplements present a more nuanced profile, showing beneficial effects for atopic dermatitis^8,9^ and cardiovascular conditions,^10^ yet simultaneously contradictory findings have been reported that challenge their universal efficacy.^11,12^ Similarly, the research on canine joint supplement usage remains equivocal, with osteoarthritis supplement studies^13,14^ yielding mixed results. Despite the lack of solid evidence for their use, supplements are thought to be commonly administered to dogs by pet owners. However, comprehensive data regarding the overall prevalence of supplement use and demographic patterns associated with use in the US remain limited. Existing research provides only preliminary insights. Freeman et al^15^ found that the overall proportion of supplement use was 10% among 107 dog owners in the US and Australia when cold-calling people listed in telephone books. Bianco et al^16^ found that supplement use among dogs with cancer diagnoses was higher compared to those without in a population of dogs referred to a tertiary care facility recruited in the waiting room of that facility. Earlier work within the Dog Aging Project (DAP) cohort using data collected in the first year of enrollment examined supplements specifically intended to support joints and found that 70% of dogs with an owner-reported diagnosis of osteoarthritis were receiving a joint supplement.^17^ These studies underscore the need for more systematic, large-scale investigations to elucidate the broader landscape of veterinary nutritional supplementation.

The DAP^18^ represents a unique opportunity to look at supplement use across a large population of pet dogs as reported by their owners. Given this, the purpose of this study was to examine the types of supplements being administered within the DAP cohort and to examine supplement use in dogs diagnosed with specific conditions that are thought to be improved with supplement administration. Additionally, dog and owner demographic variables were examined to identify potential variables that may require inclusion in future analyses. This cross-sectional study will provide context for prospective work in this cohort of dogs and inform veterinary professionals about the current state of supplement use for dogs with various conditions.

## Methods

The DAP is a community science-based project in the US that prospectively collects information from pet dog owners annually through surveys and other means.^19^ Pet owners are recruited through public outreach, including social media and word of mouth. In order to enroll their pet dog, owners are invited to complete comprehensive surveys that collect information on health conditions, environment, behavior, diet, etc. Upon completion of the surveys, the dog is enrolled, and participants will receive reminders annually to complete follow-up surveys to record changes to the information. Responses from the Health and Life Experience Survey (HLES),^20^ completed by all DAP participants at the time of enrollment, were analyzed here. The University of Washington institutional review board (IRB) deemed that recruitment of dog owners for the DAP and the administration and content of the DAP HLES are human subjects research that qualifies for category 2 exempt status (IRB ID No. 5988, effective October 30, 2018). No interactions between researchers and privately owned dogs occurred; therefore, IACUC oversight was not required. All initial diet survey information regarding supplements and the frequency of their administration as reported in the diet section of the survey, dog and owner demographic information, and health conditions reported by the owner were abstracted from HLES for dates January 1, 2020, through December 31, 2022 (2022 curated data release).^21^ If the diet section of the HLES survey was completed by the participants, the data were included in this study. In an effort to minimize selection bias, no responses were excluded. The supplement categories owners could select were bone meal, glucosamine, chondroitin, other joint, omega-3 supplements, nonoil skin, vitamins, enzyme, probiotics, fiber, urine alkalinizer, urine acidifier, taurine, antioxidants, and coenzyme Q10. Daily and less-frequent-than-daily supplement use were not mutually exclusive options. Owners responded to each frequency category with a “yes/no” option answer. As such, an owner could report both daily and less-frequent-than-daily administration of supplements, either option individually, or neither. If the owner reported either daily or less-frequent-than-daily supplement use for their dog, an additional set of questions followed, eliciting supplement type and whether that supplement was fed separately or formulated as part of the pet’s diet. If the owner reported both daily and less-frequent-than-daily supplement use, the additional set of questions appeared twice: once for the supplements given daily and once for the supplements given less frequently than daily.

Dog and owner demographic variables were summarized as counts and percentages according to the frequency of supplement administration. Dog demographic variables examined included breed status (single or mixed breed), sex class (female intact, female spayed, male intact, or male neutered), life stage (puppy, young adult, mature adult, or senior), dog primary purpose (companion, obedience, show, breeding, agility, hunting, working, service, search and rescue, or therapy), activity level (not active, moderately active, or very active), owner’s perception of the dog’s overall health (excellent, very good, good, fair, poor, or very poor), and the frequency of veterinary visits (more than once per year, about once per year, less than once per year, or never). The owner demographic variables examined were age range (categorized in roughly 10-year intervals between 18 and 75 years of age), education level, race/ethnicity, and income range. Use of supplements, both daily and less frequently than daily, was examined at the state level according to the primary state of residence.

Based on the supplement categories from the survey, specific conditions were selected based on their established relevance to supplement use as identified through prior knowledge (eg, joint supplements for arthritis). Additionally, any conditions explicitly mentioned in the dataset were reviewed and considered for inclusion. The health conditions as reported by the owner and the daily supplements that would be relevant were tabulated into tables using a combination of RStudio (version 4.4.0; R Core Team) using the R packages *dplyr* (version 1.1.4)^22^ and *tidyverse* (version 2.0.0)^23^ and Excel 2019 (Microsoft Corp) pivot tables. For example, among dogs with osteoarthritis, lameness, elbow dysplasia, and hip dysplasia diagnoses, the number and percentage of dogs receiving daily glucosamine, chondroitin, and other joint supplements were tabulated. This was done for the following combinations of supplements and health conditions: omega-3 supplements (atopy, pruritus, murmur, cardiomyopathy, valve disease, osteoarthritis, lameness, elbow dysplasia, hip dysplasia, and dementia/cognitive dysfunction), probiotics and fiber supplements (anal sacculitis, chronic idiopathic colitis, inflammatory bowel disease [IBD], and malabsorptive disorder), urine alkalinizers and acidifiers (uroliths), and antioxidants (dementia/cognitive dysfunction). Additionally, the proportion of dogs receiving separate supplements in each of the supplement categories was tabulated by life stage.

Finally, the free-text entries included in response to the survey option “other supplements” were extracted, and a qualitative analysis was conducted. Atlas. TI Web software version 8.6.2 (Scientific Software Development GmbH) was used to assign codes to all qualitative responses. Specific supplement names were coded (eg, cannabidiol [CBD], kelp, etc), and 19 category codes were additionally created, which encompassed several specific supplements with similar ingredients or supplements given for similar purposes **(**Supplementary Table S1**)**. Summary statistics of these codes were then tabulated.

### Statistical analysis

The 95% CIs for the proportions were calculated using the binomial exact method with the R package *binom*.^24^ This method was chosen over hypothesis testing because it conveys both effect size and uncertainty, relies on the reader to interpret the results within the context of their expertise, and is part of the research field’s movement away from hypothesis testing and *P* values.^25,26^

## Results

### Overall

Of the 43,517 owners who completed HLES to enroll in the DAP, 40,367 (93%) completed the diet section, which included supplement questions. Of the 40,367 owners who responded to the questions related to supplement use on the survey, 20,993 (52%) reported administering supplements to their dog either daily, less frequently than daily, or both. When examining the population of dogs receiving supplements either daily, less frequently than daily, or both as compared to those with no supplement use, single-breed dogs were more likely to receive supplements than mixed-breed dogs (11,402 of 20,386 [56%] and 9,591 of 19,981 [48%], respectively; Table 1). There was no difference in supplement use between female and male dogs (10,344 of 20,007 [52%] and 10,649 of 20,360 [52%], respectively), but intact dogs were slightly more likely to receive supplements than neutered or spayed dogs (2,364 of 4,406 [54%] and 18,629 of 35,961 [52%], respectively). Dogs with the primary purposes of hunting and companionship were least likely to receive supplements (28 of 56 [50%] and 19,821 of 38,372 [52%], respectively), whereas dogs engaged in search and rescue and agility work were most likely to receive supplements (49 of 70 [70%] and 82 of 112 [73%], respectively). Very active dogs were least likely to receive supplements (3,987 of 8,083 [49%]), whereas nonactive dogs were most likely (2,844 of 5,168 [55%]) to have supplements administered. Dogs reported to be in poorer overall health were more likely to receive supplements compared to their counterparts reported to be in better health, with supplement use increasing from 45% among dogs in excellent health to 68% for those in very poor health. Dogs that visited their veterinarian more than once per year were more likely to receive supplements (12,556 of 22,167 [57%]) than those that had never seen a veterinarian (22 of 54 [41%]). Dogs weighing over 40 kg were most likely to receive supplements (2,244 of 3,747 [60%]), whereas dogs weighing less than 10 kg were least likely to receive supplements (3,898 of 8,618 [45%]).

**Figure 1—:**
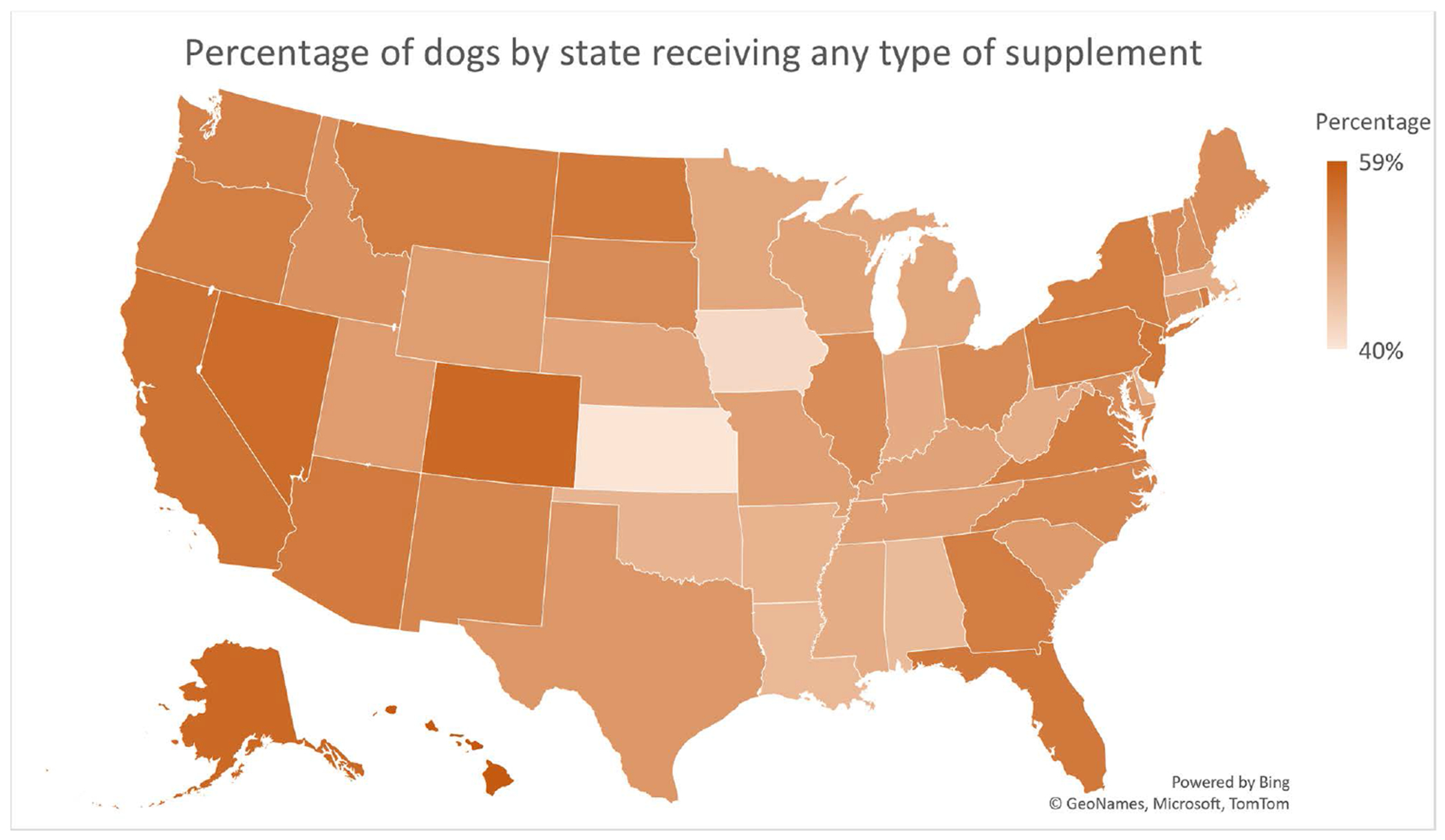
Percentage of dogs by state receiving any type of supplement at any frequency of administration as reported by owners enrolling in the Dog Aging Project, 2020 through 2022.

Examining owner demographic variables **(Table 2)**, there was a slight trend for older owners to be more likely to give supplements, with 1,011 of 1,894 (53%) owners older than 75 years of age giving supplements as compared to 359 of 750 (48%) owners 18 to 24 years old giving supplements. Owners with fewer years of education were more likely to give supplements than owners with more education. There were no large differences by owner income and only small differences by owner self-identified race. In general, dogs in the Midwestern states and those adjacent to the Ohio and Mississippi Rivers reported lower supplement administration than those along the coasts or in the Rocky Mountain areas **(Figure 1)**.

Among all dogs receiving supplements of any kind in the DAP at the time of the study (20,993), the supplements most commonly received were omega-3 fatty acids (11,934 [57%]), glucosamine (11,810 [56%]), chondroitin (9,548 [45%]), vitamins (7,948 [38%]), probiotics (7,710 [37%]), fiber (5,798 [28%]), other joint (5,247 [25%]), antioxidants (4,523 [22%]), taurine (3,036 [14%]), enzyme (2,952 [14%]), bone meal (2,797 [13%]), coenzyme Q10 (1,772 [8%]), urine alkalinizer (958 [5%]), and urine acidifier (884 [4%]).

### Daily and less frequent supplement use

**Figure 2—:**
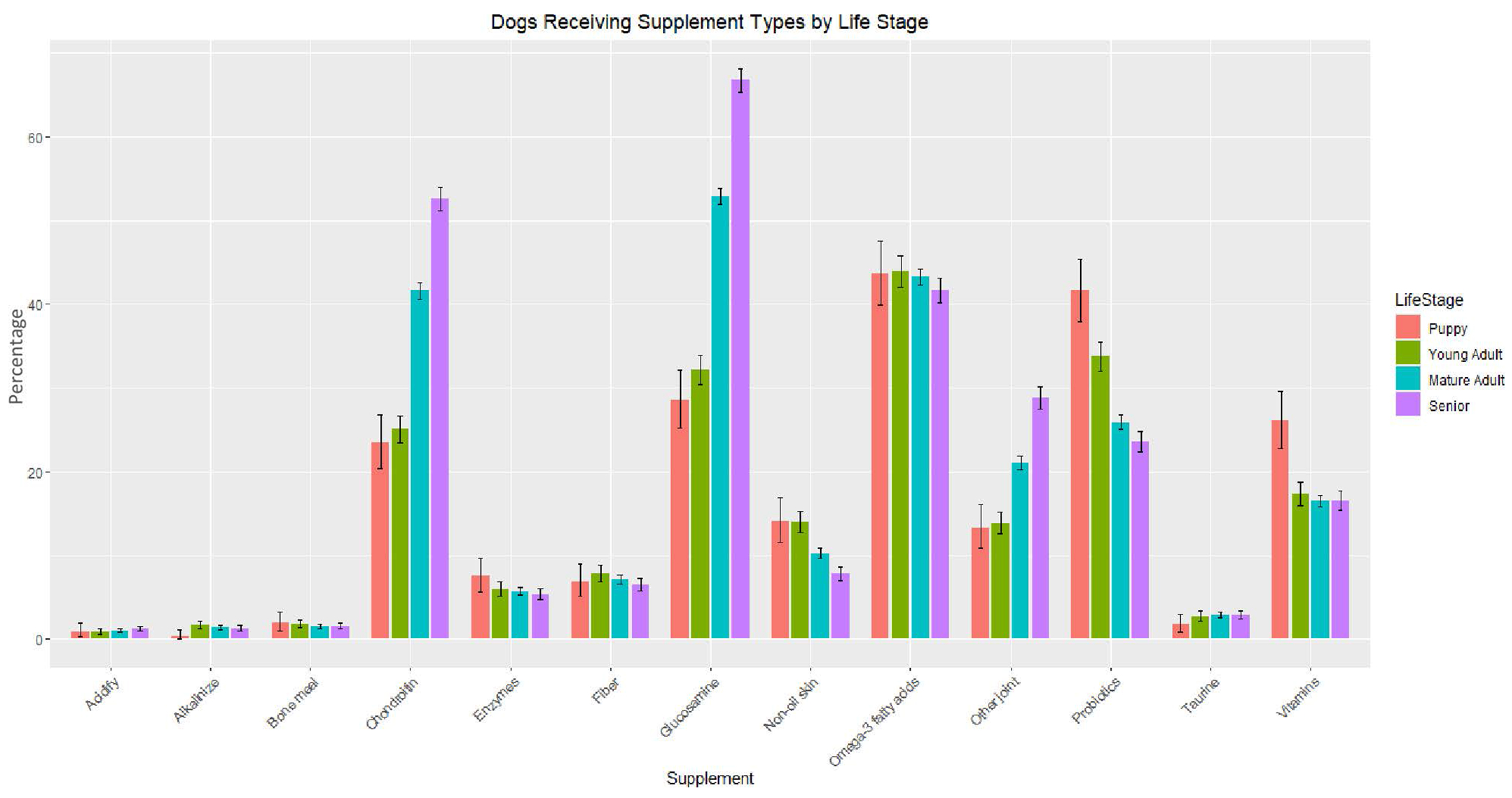
Percentage of dogs receiving different types of supplements, stratified by life stage, as reported by owners enrolling in the Dog Aging Project, 2020 through 2022.

Daily administration of supplements was reported by 18,569 of 40,367 owners (46%). Only 4,588 of 40,367 owners (11%) reported less frequent use of supplements. Dog demographic variables showed more variability across their levels compared to owner demographic variables. Single-breed dogs were more likely to receive supplements compared to mixed-breed dogs (10,195 of 20,371 [50%] and 8,352 of 19,958 [42%], respectively; Supplementary Table S2). There was no difference in the use of supplements between female and male dogs (9,137 of 19,988 [46%] and 9,410 of 20,341 [46%], respectively), but intact dogs were slightly more likely to receive daily supplements compared to neutered dogs (2,080 of 4,406 [47%] and 16,467 of 35,923 [46%], respectively). Puppies were the least likely age group to receive daily supplements (682 of 1,862 [37%]), whereas senior dogs were most likely to receive daily supplements (4,587 of 7,463 [61%]). However, when examining supplement type over life stage, individual supplement types were administered differently to different age groups **(Figure 2)**. Chondroitin, glucosamine, and other joint supplements were administered to older dogs more than younger dogs, whereas probiotics, vitamins, and nonoil skin supplements were administered to younger dogs more. Omega-3 fatty acids were administered similarly across the life stages. Dogs whose primary purpose was agility, breeding, show, or service were more likely to receive supplements (77 of 112 [69%], 35 of 61 [57%], 40 of 70 [57%], and 215 of 387 [56%], respectively) compared to companion dogs and hunting or other working dogs (17,504 of 38,335 [46%], 57 of 122 [47%], and 23 of 56 [41%], respectively). The proportion of dogs receiving supplements increased with decreasing activity level, so “not active” dogs received daily supplements the most (2,553 of 5,167 [49%]). There was an inverse relationship between the owner-reported health status of the dogs and supplement use, with dogs in excellent health being less likely to receive supplements than dogs in fair, poor, or very poor health

As owners reported higher ages for themselves, the percentages of daily supplement use provided to their dogs also increased, with 285 of 750 (38%) 18- to 24-year-olds using daily supplements and 4,331 of 8,607 (50%) 65- to 74-year-olds using daily supplements for their dogs **(**Supplementary Table S3**)**. Owner education level, race/ethnicity, and income did not vary substantially across supplement use groups. Geographically, Iowa had the lowest percentage of dogs receiving daily supplements (123 of 440 [28%]), whereas North Dakota had the highest (32 of 68 [47%]; Supplementary Figure S1).

**Figure 3—:**
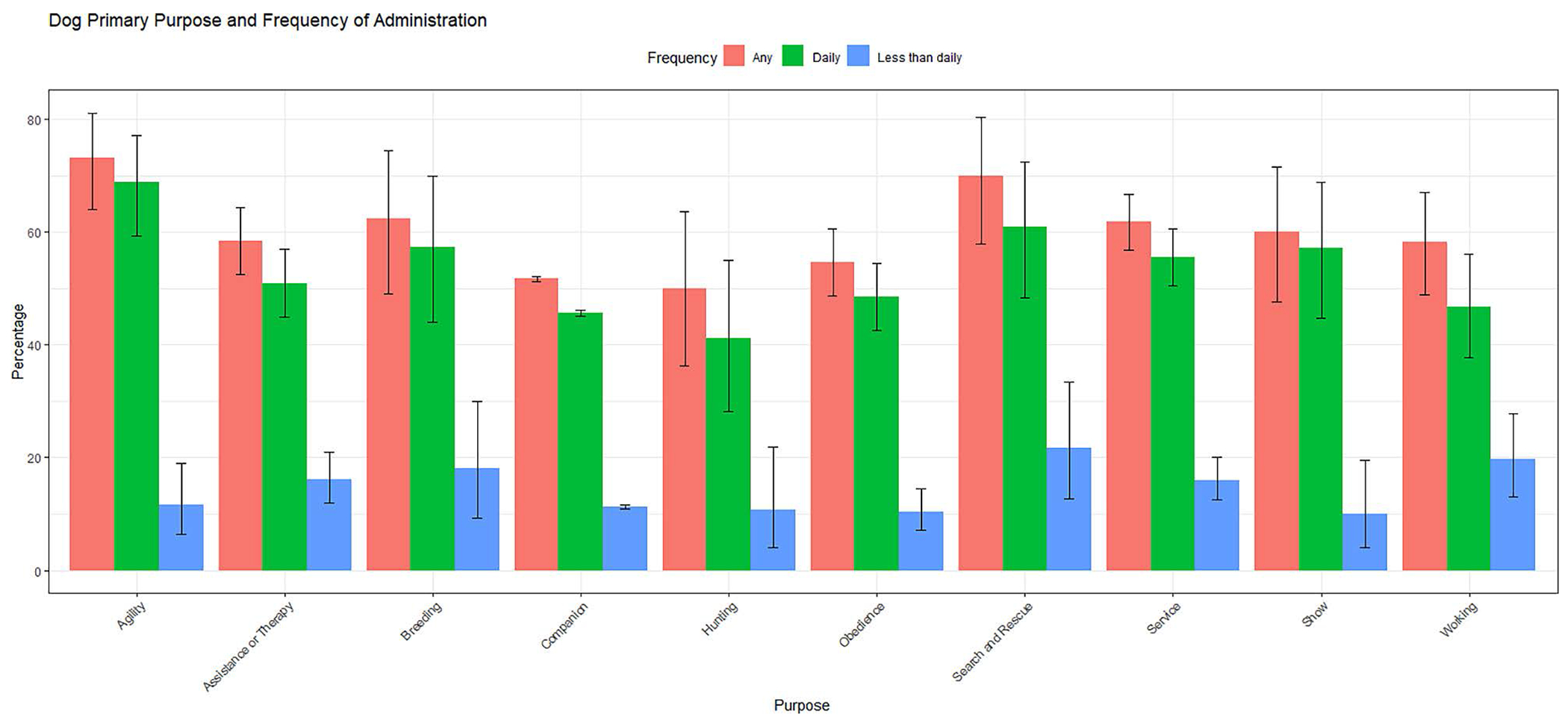
Percentage of dogs receiving any supplement, daily supplements, and less-frequent-than-daily supplements, stratified by dog primary purpose, as reported by owners enrolling in the Dog Aging Project, 2020 through 2022.

Less-frequent-than-daily supplement use had very similar results to daily supplement use. Dog breed status, sex, and neuter status had similar results **(**Supplementary Table S4**)**. Working, breeding, and service dogs were most likely to receive supplements less frequently than daily, and this can be compared to any supplement use and daily supplement use in Figure 3. The notable exceptions to this similarity were owner demographic variables: less frequent supplement use was highest among younger owners, was increasingly lower with increasing owner age **(**Supplementary Table S5**)**, was highest among those reporting the lowest household income, and was slightly lower with increasing income when compared to no supplement use. Geographically, Iowa again had the lowest reported percentages of less-frequent-than-daily supplement use (Supplementary Figure S1).

### Daily supplement types among the health conditions

**Figure 4—:**
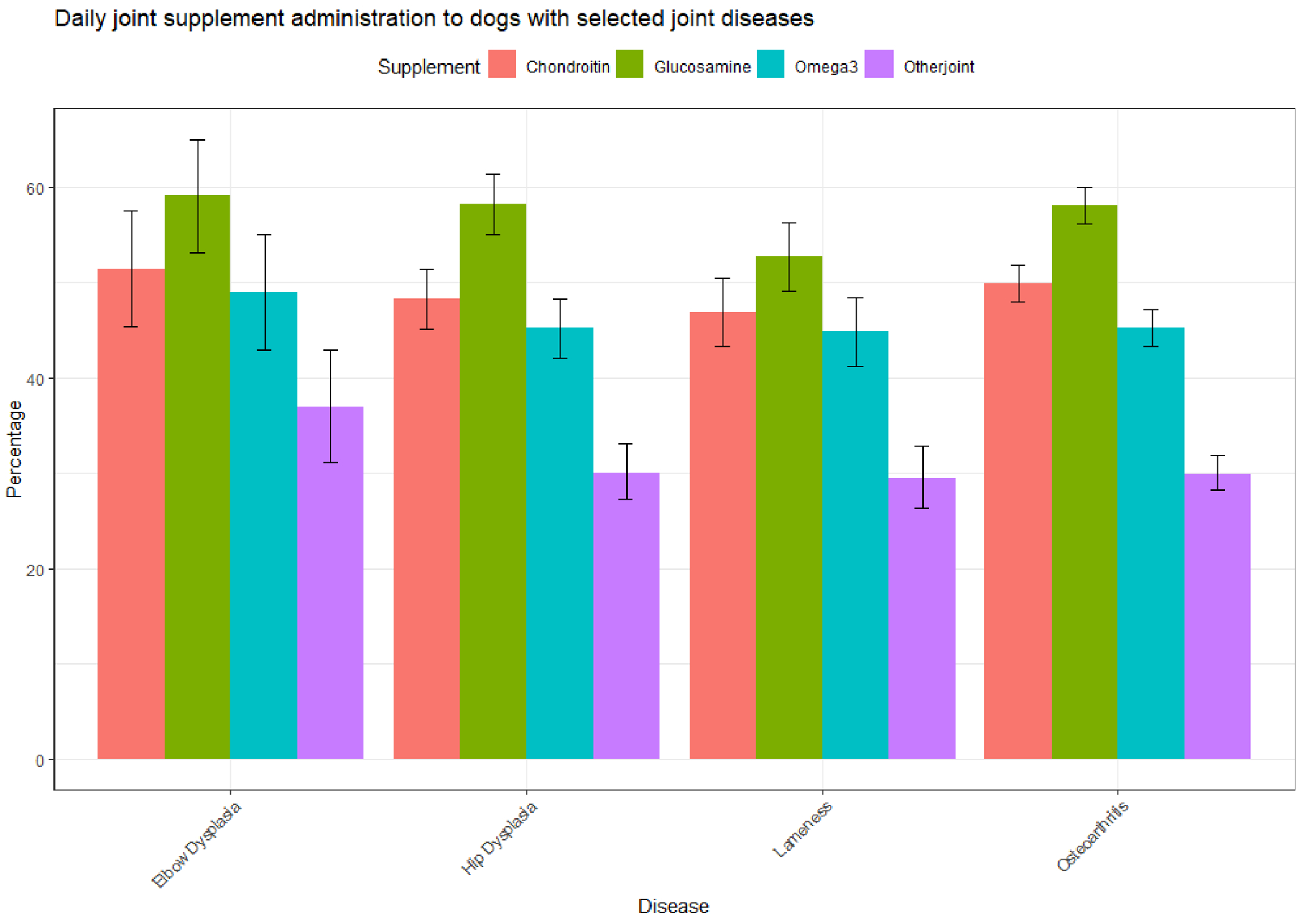
Percentage of dogs with specified joint diseases receiving joint supplements as reported by owners enrolling in the Dog Aging Project, 2020 through 2022.

Dogs diagnosed with orthopedic conditions were most frequently receiving supplements daily (as opposed to less than daily). Dogs diagnosed with reproductive conditions were most commonly reported to be receiving supplements on a less-frequent-than-daily basis. While omega-3 fatty acid supplements were most commonly reported overall, they were generally provided to dogs with orthopedic conditions, with elbow and hip dysplasia being the most frequent diagnoses (134 of 274 [49%] and 446 of 988 [45%], respectively; Figure 4). Glucosamine was the most commonly used joint supplement across the selected joint conditions (osteoarthritis, lameness, elbow dysplasia, and hip dysplasia). Dogs diagnosed with elbow dysplasia received all joint supplements the most compared to the other joint conditions.

Omega-3 fatty acid supplements were provided to 39% of dogs with atopy and 36% of dogs with skin pruritus. Forty-five percent of dogs with murmurs and valve disease both received omega-3 fatty acid supplements daily. Probiotics were given to 23% of dogs with anal sacculitis, 34% of dogs with chronic idiopathic colitis, 38% of dogs with IBD, and 48% of dogs with malabsorptive intestinal disease. Fiber supplementation was provided to 21% of dogs with anal sacculitis, 18% of dogs with chronic idiopathic colitis, 23% of dogs with IBD, and 20% of dogs with malabsorptive disorder. Of 240 dogs with reported dementia, 91 (38%) received omega-3 supplementation, and 49 (20%) received antioxidants.

### Qualitative analysis and coding of “other” free text

A total of 3,995 free-text responses were recorded. Of those, 143 were blank or not applicable responses, and 156 included information about something other than supplement administration. These 156 responses included food items (green beans, chicken, etc) and prescription medications in pill or powder form (including gabapentin, thyroxine, carprofen, grapiprant, meloxicam, famotidine, phenylpropanolamine, omeprazole, pimobendan, furosemide, benazepril, and tylosin). Therapeutic diets were also reported, some appropriately listed as including supplements that the survey had previously listed (eg, glucosamine and chondroitin as part of therapeutic joint diets or fiber and probiotics as part of gastrointestinal diets), and other therapeutic diets that are more frequently considered elimination-type diets (eg, hydrolyzed protein diets) were also listed.

Of the remaining responses, 784 (20%) were found to be supplements that already existed in the survey list. The remaining 1,445 responses were grouped into the following categories: CBD or hemp (518), turmeric (274), liver support (including S-adenosyl-L-methionine and silybin; 218), dental supplements (which included chews, powders, and liquids; 188), kelp powder or flakes (129), cranberry (117), calming (102), Chinese herb (69), anal gland (55), cardiac (53), eye (52), melatonin (49), seaweed (39), nonomega algae (such as spirulina, chlorella, etc; 38), apple cider vinegar (27), prebiotic (25), and coprophagia prevention (18). Other supplements listed were bee pollen (20), bladder support (non–pH modifying and non–cranberry extract), egg shells, colostrum, telomeres, prions, and montmorillonite clay. Some owners reported giving items that may have deleterious effects, such as garlic supplements (53) and aspirin (7), and feeding diatomaceous earth (3).

## Discussion

The use of supplements was reported in 52% of the dogs included in this study, which is higher than reported in previous reports.^15,27^ However, this cohort consists of owners who voluntarily complete lengthy surveys about their pet’s health and lifestyle for no compensation and may not be representative of the total US population of dogs or dog owners. Previous supplement use prevalence estimates involved random phone calls to anyone who could be a pet owner (or not)^15^ and participants recruited from the waiting room of a tertiary care facility.^16^

Comparing demographics between any, daily, and less frequent supplement use provides mostly similarities but a few differences. Dog breed status, sex, neuter status, life stage, and health status did not differ between those receiving supplements daily or less frequently than daily nor did education level. However, owner income and age did show different trends between the frequencies of supplement administration. Daily supplementation showed no differences across owner income levels, whereas less-than-daily supplementation was highest among the lower income levels and decreased with increasing owner income. Additionally, the trends for owner age were in opposite directions depending on whether the supplements were given daily or less frequently than daily, with daily supplement use increasing and less frequent supplement use decreasing with higher owner age. Perhaps as owners age and begin to take daily supplements and medications, they are inclined to administer supplements to their pets more frequently, or perhaps the fact that older owners also tend to own older dogs may be the contributing factor to this difference, and further research should be done.^28^ Supplements of all types tended to be administered more often when less-optimal general health was reported. This might suggest a tendency for owners to respond to health issues by giving supplements. However, certain types of supplements were more likely to be given to older dogs (joint supplements of any type), some were more likely to be administered to younger dogs (probiotics and vitamins), and some were given equally to all life stages (omega-3 fatty acids). Geographically, there were no striking trends with supplement use like there were with differences in diet type choices observed in this same population over the same time period.^28^ In this population of dogs, single-breed dogs are both more likely to receive supplements and more likely to consume raw diets (both commercial and home prepared). The reasons for the differences in both diet choices and supplement administration may be related, but determining owner motivations would require special IRB approval. Further research in this area is necessary.

Looking at the use of supplements within certain owner-reported conditions provides valuable information to veterinary practitioners, researchers, industry, and owners. Dogs with orthopedic diseases were most likely to receive supplements compared to other conditions. While the efficacy of some of these supplements has not been demonstrated, with mixed scientific evidence depending on the supplement type,^14^ the high proportion of administration of supplements for this disease group demonstrates owners’ willingness to treat a chronic disease. Additionally, while joint supplements were commonly utilized, the prevalence of urine-modifying supplements was notably lower. A relatively low proportion (25%) of dogs with uroliths received any type of urine-modifying supplement as identified by their owners.

Therapeutic diets, which include supplements and vitamins as ingredients in their commercial preparation, and, conversely, whole-food ingredients that are used as supplements (eg, yogurt as a probiotic) were variably reported by owners responding to the DAP survey supplement questions. Some owners also included topical over-the-counter items, like eye lubricants, as supplements, and several mentioned these items along with ingested supplements (eg, “Optixcare and Angel Eyes Powder”), suggesting that owners group these items together by their intended effect. Some prescription medications were also misidentified as supplements and were listed either singularly (especially the prescription drug that contains phenylpropanolamine, Proin) or were included with other supplements. Grouping them together was particularly common for joint support medications (eg, a list of glucosamine, chondroitin, and meloxicam). While the veterinary industry and food manufacturers have legal definitions of when an item is a drug, those definitions are not universally understood by pet owners. Owners consider items to be supplements that are actually drugs, and they consider supplements to be drugs when they are not. Given this information, there is evidence for misclassification error within this supplement dataset since some owners misreport or misunderstand what a supplement is. However, it is expected that this misclassification is equal across all groups within this cohort.

Furthermore, when considering the frequency of dosing of supplements, some supplements are recommended to be given daily, whereas others may not require daily treatment (eg, calming supplements given only in certain situations). While the survey did allow for owners to choose both daily and less-frequent-than-daily options (ie, a dog receiving both daily and less-frequent-than-daily supplements could be represented as such), it is important to interpret the differences between daily and less-frequent-than-daily administration in the context of which supplements may be recommended to be administered at different frequencies.

Future work with this dataset will require researchers to inspect these free-text responses to ensure that supplements as defined by owners are truly supplements. Surveys designed to assess supplement usage in dogs in the future may also benefit from a strict inclusion definition and examples so that owners have greater clarity of what information should be provided.

When owners listed the reason for the use of some supplements, the specificity with which they assigned uses varied. For instance, CBD, kelp, seaweed, and algae were frequently associated with several intended uses, whereas glucosamine was generally given with the intention to treat arthritis or other joint conditions.

Some of the supplements identified in the free-text qualitative analysis described here have been added as responses to the DAP diet survey that was deployed on January 1, 2023, as part of the comprehensive diet questionnaire. The additional supplement categories include CBD, turmeric, liver support, immune support, and allergy support. Furthermore, the frequency of administration variable has been changed to be a daily equivalent frequency (ranging from monthly to several times per day) rather than just daily or less frequent. This will benefit researchers as the frequency of administration can be stratified in analyses to provide more fidelity.

With the sample size of the DAP, small differences across demographic levels are often statistically significant, but their practical relevance varies by audience. A general practitioner, for example, may interpret findings on arthritis supplement use differently depending on their perspective on supplement efficacy, and small differences in the demography would not necessarily be important. Meanwhile, those minor differences across populations may be highly relevant to an industry veterinarian. The data have been presented as transparently as possible to allow readers to interpret it in the context of their expertise and relevance to their practice area.

For researchers, the DAP cohort is well positioned to be able to contribute to efficacy research. While randomized controlled trials are considered to be the gold standard for investigating the efficacy of supplements (or the effect of treatments), well-designed population-based studies can approximate the magnitude of effects while also being cheaper and generalizable to broader population demographics.^29,30^ The large population of dogs included in the DAP cohort also have information collected from their owners annually, so temporal relationships between supplement use and health outcomes can be established.

For clinicians and pet care teams, the fact that so many medications were confused as supplements, and that supplement use was so common in this cohort, should be an indication to discuss supplements in more depth with owners. Helping owners distinguish between supplements and medications can be important since supplements are not required to demonstrate safety or efficacy through clinical trials nor are they allowed to make health claims. While some supplements may offer health benefits, and indeed may be veterinarian recommended, clarifying the difference between a supplement and a medication helps owners to make informed decisions. Given the prevalence of use within this cohort, and in previous studies, supplements are very commonly used and can be an opportunity to engage owners.

Ultimately, the key takeaway appears to be this: a large proportion of owners report supplement use, with the most common supplements reported being omega-3 fatty acids and joint supplements. However, there are many supplements that do not conform to the categories presented as part of the survey, which demonstrates the breadth of conditions for which supplements are marketed. Some owners seem to confuse prescription medications with supplements. Supplements appear to be given more often to older dogs that visit the veterinarian less frequently than once per year; however, a good amount of apparently healthy dogs are receiving supplements.

## Supplementary Material

Supplementary Table S3

Supplementary Table S5

Supplementary Table S4

Supplementary Table S2

Supplementary Table S1

Supplementary Figure S1

Supplementary materials are posted online at the journal website: avmajournals.avma.org.

## Figures and Tables

**Table 1—:** Dog demographic variables and any supplement administration to pet dogs as reported by their owners as part of the Dog Aging Project baseline survey, 2020 through 2022.

**Variable**	**No supplement use**	**%**	**95% CI**	**Any supplement use**	**%**	**95% CI**	**Total**
Breed status							
Mixed breed	10,390	52	51–53	9,591	48	47–49	19,981
Purebred	8,984	44	43–45	11,402	56	55–57	20,386
Sex							
Female	9,663	48	48–49	10,344	52	51–52	20,007
Male	9,711	48	47–48	10,649	52	52–53	20,360
Neuter status							
Intact	2,042	46	45–48	2,364	54	52–55	4,406
Neutered	17,332	48	48–49	18,629	52	51–52	35,961
Life stage							
Puppy	1,088	58	56–61	774	42	39–44	1,862
Young adult	4,811	58	57–59	3,476	42	41–43	8,287
Mature adult	10,959	48	48–49	11,755	52	51–52	22,714
Senior	2,485	33	32–34	4,979	67	66–68	7,464
Dog primary purpose							
Companion	18,551	48	48–49	19,821	52	51–52	38,372
Obedience	131	45	39–51	158	55	49–61	289
Show	28	40	28–52	42	60	48–72	70
Breeding	23	38	26–51	38	62	49–74	61
Agility	30	27	19–36	82	73	64–81	112
Hunting	28	50	36–64	28	50	36–64	56
Working	51	42	33–51	71	58	49–67	122
Service	148	38	33–43	239	62	57–67	387
Search and rescue	21	30	20–42	49	70	58–80	70
Assistance or therapy	116	42	36–48	163	58	52–64	279
Activity level							
Very active	4,106	51	50–52	3,987	49	48–50	8,093
Moderately active	12,944	48	47–48	14,162	52	52–53	27,106
Not active	2,324	45	44–46	2,844	55	54–56	5,168
General health							
Excellent	10,789	55	54–55	8,988	45	45–46	19,777
Very good	6,129	44	44–45	7,698	56	55–57	13,827
Good	1,845	37	36–39	3,114	63	61–64	4,959
Fair	498	34	31–36	972	66	64–69	1,470
Poor	95	34	29–40	182	66	60–71	277
Very poor	18	32	20–45	39	68	55–80	57
Vet visit frequency							
More than once/year	9,611	43	43–44	12,556	57	56–57	22,167
About once/year	9,250	54	53–54	8,011	46	46–47	17,261
Less than once/year	481	54	51–58	404	46	42–49	885
Never	32	59	45–72	22	41	28–55	54
Dog size (kg)^a^							
0–9.9	4,720	55	54–56	3,898	45	44–46	8,618
10–19.9	4,104	50	49–51	4,047	50	49–51	8,151
20–29.9	5,740	48	47–49	6,311	52	51–53	12,051
30–39.9	3,271	42	41–43	4,484	58	57–59	7,755
40+	1,503	40	39–42	2,244	60	58–61	3,747

a Forty-five dogs did not have a weight reported and are not included in these weight categories.

**Table 2—:** Owner demographic variables and any supplement administration to pet dogs as reported by their owners as part of the Dog Aging Project baseline survey, 2020 through 2022.

**Variable**	**No supplement use**	**%**	**95% CI**	**Any supplement use**	**%**	**95% CI**	**Total**
Owner age range							
18–24	391	52	48–56	359	48	44–52	750
25–34	2,716	50	48–51	2,741	50	49–52	5,457
35–44	3,088	49	47–50	3,256	51	50–53	6,344
45–54	3,580	50	48–50	3,645	50	49–52	7,225
55–64	4,837	48	47–49	5,245	52	51–53	10,082
65–74	3,879	45	44–46	4,736	55	54–56	8,615
> 75	883	47	44–49	1,011	53	51–56	1,894
Owner max education							
High school or less	483	48	44–51	533	52	49–56	1,016
Trade, technical, or vocational	475	45	42–48	583	55	52–58	1,058
Some college, no degree	1,742	46	45–48	2,023	54	52–55	3,765
Associate degree	1,133	46	44–48	1,356	54	53–56	2,489
Bachelor’s degree	6,595	47	46–48	7,395	53	52–54	13,990
Master’s degree	5,519	49	48–50	5,764	51	50–52	11,283
Professional degree	1,799	50	48–51	1,814	50	49–52	3,613
Doctorate degree	1,628	52	50–53	1,525	48	47–50	3,153
Owner race							
White	18,421	48	48–49	19,779	52	51–52	38,200
Black or African American	240	47	42–51	272	53	49–58	512
Asian	630	42	40–45	861	58	55–60	1,491
American Indian	243	44	40–48	310	56	52–60	553
Hispanic	787	48	46–51	836	52	49–54	1,623
Owner income range							
< $20,000	344	46	43–50	398	54	50–57	742
$20,000–39,999	1,107	47	45–49	1,267	53	51–55	2,374
$40,000–59,999	1,868	49	47–50	1,965	51	50–53	3,833
$60,000–79,999	2,109	47	46–49	2,340	53	51–54	4,449
$80,000–99,999	2,100	49	47–50	2,216	51	50–53	4,316
$100,000–119,999	2,144	48	47–49	2,325	52	51–53	4,469
$120,000–139,999	1,546	48	47–50	1,653	52	50–53	3,199
$140,000–159,999	1,278	48	46–50	1,372	52	50–54	2,650
$160,000–179,999	930	51	49–54	879	49	46–51	1,809
≥$180,000	3,722	49	48–50	3,879	51	50–52	7,601

## References

[1] Dietary Supplement Health and Education Act of 1994. NIH Office of Dietary Supplements. October 25, 1994. Accessed November 26, 2024. https://ods.od.nih.gov/About/DSHEA_Wording.aspx

[2] Finno CJ. Veterinary pet supplements and nutraceuticals. Nutr Today. 2020;55(2):97–101. doi:10.1097/NT.0000000000000399 33446942 PMC7802882

[3] Dzanis DA. Understanding regulations affecting pet foods. Top Companion Anim Med. 2008;23(3):117–120. doi:10.1053/j.tcam.2008.04.002 18656837

[4] Fritsch DA, Wernimont SM, Jackson MI, MacLeay JM, Gross KL. A prospective multicenter study of the efficacy of a fiber-supplemented dietary intervention in dogs with chronic large bowel diarrhea. BMC Vet Res. 2022;18(1):244. doi:10.1186/s12917-022-03302-8 35751062 PMC9229818

[5] Alves JC, Santos A, Jorge P, Pitães A. The use of soluble fibre for the management of chronic idiopathic large-bowel diarrhoea in police working dogs. BMC Vet Res. 2021;17(1):100. doi:10.1186/s12917-021-02809-w 33653329 PMC7923632

[6] White R, Atherly T, Guard B, Randomized, controlled trial evaluating the effect of multi-strain probiotic on the mucosal microbiota in canine idiopathic inflammatory bowel disease. Gut Microbes. 2017;8(5):451–466. doi:10.1080/19490976.2017.1334754 28678609 PMC5628651

[7] Jensen AP, Bjørnvad CR. Clinical effect of probiotics in prevention or treatment of gastrointestinal disease in dogs: a systematic review. J Vet Intern Med. 2019;33(5): 1849–1864. doi:10.1111/jvim.15554 31313372 PMC6766488

[8] Olivry T, DeBoer DJ, Favrot C, Treatment of canine atopic dermatitis: 2015 updated guidelines from the International Committee on Allergic Diseases of Animals (ICADA). BMC Vet Res. 2015;11(1):210. doi:10.1186/s12917-015-0514-6 26276051 PMC4537558

[9] Popa I, Pin D, Remoué N, Analysis of epidermal lipids in normal and atopic dogs, before and after administration of an oral omega-6/omega-3 fatty acid feed supplement: a pilot study. Vet Res Commun. 2011;35(8):501–509. doi:10.1007/s11259-011-9493-7 21786009

[10] Freeman LM. Beneficial effects of omega-3 fatty acids in cardiovascular disease. J Small Anim Pract. 2010;51(9):462–470. doi:10.1111/j.1748-5827.2010.00968.x 20673293

[11] Mueller RS, Fettman MJ, Richardson K, Plasma and skin concentrations of polyunsaturated fatty acids before and after supplementation with n-3 fatty acids in dogs with atopic dermatitis. Am J Vet Res. 2005;66(5):868–873. doi:10.2460/ajvr.2005.66.868 15934614

[12] Mueller RS, Fieseler KV, Fettman MJ, Effect of omega-3 fatty acids on canine atopic dermatitis. J Small Anim Pract. 2004;45(6):293–297. doi:10.1111/j.1748-5827.2004.tb00238.x 15206474

[13] Comblain F, Serisier S, Barthelemy N, Balligand M, Henrotin Y. Review of dietary supplements for the management of osteoarthritis in dogs in studies from 2004 to 2014. J Vet Pharmacol Ther. 2016;39(1):1–15. doi:10.1111/jvp.12251 26205697

[14] Barbeau-Grégoire M, Otis C, Cournoyer A, Moreau M, Lussier B, Troncy E. A 2022 systematic review and meta-analysis of enriched therapeutic diets and nutraceuticals in canine and feline osteoarthritis. Int J Mol Sci. 2022;23(18):10384. doi:10.3390/ijms231810384 36142319 PMC9499673

[15] Freeman LM, Abood SK, Fascetti AJ, Disease prevalence among dogs and cats in the United States and Australia and proportions of dogs and cats that receive therapeutic diets or dietary supplements. J Am Vet Med Assoc. 2006;229(4):531–534. doi:10.2460/javma.229.4.531 16910851

[16] Bianco AV, Abood S, Mutsaers A, Woods JP, Coe JB, Verbrugghe A. Unconventional diets and nutritional supplements are more common in dogs with cancer compared to healthy dogs: an online global survey of 345 dog owners. Vet Comp Oncol. 2020;18(4):706–717. doi:10.1111/vco.12599 32304175

[17] Hoffman JM, Tolbert MK, Promislow DEL; Dog Aging Project Consortium. Demographic factors associated with joint supplement use in dogs from the Dog Aging Project. Front Vet Sci. 2022;9:906521. doi:10.3389/fvets.2022.906521 35958315 PMC9361857

[18] Creevy KE, Akey JM, Kaeberlein M, Promislow DEL; Dog Aging Project Consortium. An open science study of ageing in companion dogs. Nature. 2022;602(7895):51–57. doi:10.1038/s41586-021-04282-9. Published correction appears in Nature. 2022;608(7924):E33. doi:10.1038/s41586-022-05179-x 35110758 PMC8940555

[19] Creevy KE, Akey JM, Kaeberlein M, Promislow DEL; Dog Aging Project Consortium. Author correction: an open science study of ageing in companion dogs. Nature. 2022;608(7924):E33. doi:10.1038/s41586-022-05179-x 35941194

[20] Survey_instruments/HLES at master: dogagingproject/dataRelease. Github. Accessed March 6, 2025. https://github.com/dogagingproject/dataRelease/tree/master/Survey_Instruments/HLES

[21] Welcome to Terra community workbench. Terra. Accessed March 20, 2025. https://app.terra.bio/#workspaces/dap-curated-download-2021/Dog%20Aging%20Project%20-%202022%20Curated%20Data%20Release

[22] Wickham H, François R, Henry L, Müller K, Vaughan D. dplyr: A Grammar of Data Manipulation. 2023. Accessed September 5, 2025. https://dplyr.tidyverse.org

[23] Wickham H, Averick M, Bryan J, Welcome to the tidyverse. J Open Source Softw. 2019;4(43):1686. doi:10.21105/joss.01686

[24] binom: binomial confidence intervals for several parameterizations. Comprehensive R Archive Network. Accessed March 19, 2025. https://CRAN.R-project.org/package=binom

[25] Wasserstein RL, Lazar NA. The ASA statement on p-values: context, process, and purpose. Am Stat. 2016;70(2):129–133. doi:10.1080/00031305.2016.1154108

[26] Fortier LA. Moving away from an overreliance on P values. J Am Vet Med Assoc. 2025;263(2):143. doi:10.2460/javma.263.2.143 39826251

[27] Hudson E, Hollar R, Wernimont S. Dietary supplement use among older dogs. FASEB J. 2021;35(S1). doi:10.1096/fasebj.2021.35.s1.04564

[28] O’Brien JS, Tolbert MK, Dog Aging Project Consortium, Ruple A. Dog and owner demographics impact dietary choices in Dog Aging Project cohort. J Am Vet Med Assoc. 2024;262(12):1676–1685. doi:10.2460/javma.24.05.0358 39142333 PMC12093940

[29] Concato J, Shah N, Horwitz RI. Randomized, controlled trials, observational studies, and the hierarchy of research designs. N Engl J Med. 2000;342(25):1887–1892. doi:10.1056/NEJM200006223422507 10861325 PMC1557642

[30] Benson K, Hartz AJ. A comparison of observational studies and randomized, controlled trials. N Engl J Med. 2000;342(25):1878–1886. doi:10.1056/NEJM200006223422506 10861324

