# Supplementary material for: Supplement use is common in Dog Aging Project participants, especially among dogs with orthopedic conditions, and varies by life stage
Source: Am J Vet Res. Author manuscript; Available in PMC 2026 Jul 11. (PMC13355649; doi:10.2460/ajvr.25.06.0217)
Supplement: Supplementary Table S3 [file NIHMS2157768-supplement-Supplementary_Table_S3.pdf]

Supplementary Table S3: Owner demographic variables and daily supplementation as reported by owners as part of the Dog Aging Project initial survey, 2020-2022

Variable	Levels	No daily sup.	%	95% CI	Daily sup. use	%	95% CI	Total
Owner Age Range	18-24	465	62%	58-65	285	38%	35-42	750
	25-34	3198	59%	57-60	2254	41%	40-43	5452
	35-44	3461	55%	53-56	2878	45%	44-47	6339
	45-54	4042	56%	55-57	3176	44%	43-45	7218
	55-64	5366	53%	52-54	4704	47%	46-48	10070
	65-74	4276	50%	49-51	4331	50%	49-51	8607
	>75	974	51%	49-54	919	49%	46-51	1893
Owner Max Education	HS or less	535	53%	50-56	480	47%	44-50	1015
	Trade, technical, or vocational	538	51%	48-54	519	49%	46-52	1057
	Some college, no degree	1965	52%	51-54	1797	48%	46-49	3762
	Associate Degree	1291	52%	50-54	1198	48%	46-50	2489
	Bachelor's Degree	7466	53%	53-54	6510	47%	46-47	13976
	Master's Degree	6191	55%	54-56	5080	45%	44-46	11271
	Professional Degree	2003	56%	54-57	1605	44%	43-46	3608
	Doctorate Degree	1793	57%	55-59	1358	43%	41-45	3151
Owner Race	White	20654	54%	54-55	17510	46%	45-46	38164
	Black or African American	283	55%	51-60	228	45%	40-49	511
	Asian	739	50%	47-52	750	50%	48-53	1489
	American Indian	282	51%	47-55	272	49%	45-53	554
	Hispanic	896	55%	53-58	728	45%	42-47	1624
Owner Income Range	<$20,000	401	54%	50-58	341	46%	42-50	742
	$20,000-39,999	1286	54%	52-56	1085	46%	44-48	2371

	$40,000-59,999	2114	55%	54-57	1718	45%	43-46	3832
	$60,000-79,999	2392	54%	52-55	2054	46%	45-48	4446
	$80,000-99,999	2370	55%	53-56	1945	45%	44-47	4315
	$100,000-119,999	2407	54%	52-55	2055	46%	45-48	4462
	$120,000-139,999	1733	54%	52-56	1463	46%	44-48	3196
	$140,000-159,999	1429	54%	52-56	1220	46%	44-48	2649
	$160,000-179,999	1022	57%	54-59	785	43%	41-46	1807
	$180,000 or more	4123	54%	53-55	3465	46%	45-47	7588
