# Supplementary material for: Supplement use is common in Dog Aging Project participants, especially among dogs with orthopedic conditions, and varies by life stage
Source: Am J Vet Res. Author manuscript; Available in PMC 2026 Jul 11. (PMC13355649; doi:10.2460/ajvr.25.06.0217)
Supplement: Supplementary Table S5 [file NIHMS2157768-supplement-Supplementary_Table_S5.pdf]

Supplementary Table S5: Owner demographic variables and less frequent than daily supplementation as reported by owners as part of the Dog Aging Project initial survey, 2020-2022

Variable	Levels	No sup.	%	95% CI	Less than daily sup.	%	95% CI	Total
Owner Age Range	18-24	619	83%	80-85	131	17%	15-20	750
	25-34	4647	85%	84-86	805	15%	14-16	5452
	35-44	5554	88%	87-88	785	12%	12-13	6339
	45-54	6379	88%	88-89	839	12%	11-12	7218
	55-64	9018	90%	89-90	1052	10%	10-11	10070
	65-74	7807	91%	90-91	800	9%	9-10	8607
	>75	1721	91%	90-92	172	9%	8-10	1893
Owner Max Education	HS or less	908	89%	87-91	107	11%	9-13	1015
	Trade, technical, or vocational	922	87%	85-89	135	13%	11-15	1057
	Some college, no degree	3289	87%	86-88	473	13%	12-14	3762
	Associate Degree	2201	88%	87-90	288	12%	10-13	2489
	Bachelor's Degree	12324	88%	88-89	1652	12%	11-12	13976
	Master's Degree	10045	89%	89-90	1226	11%	10-11	11271
	Professional Degree	3227	89%	88-90	381	11%	10-12	3608
	Doctorate Degree	2829	90%	89-91	322	10%	9-11	3151
Owner Race	White	33921	89%	89-89	4243	11%	11-11	38164
	Black or African American	429	84%	80-87	82	16%	13-20	511
	Asian	1244	84%	82-85	245	16%	15-18	1489
	American Indian	481	87%	84-90	73	13%	10-16	554
	Hispanic	1406	87%	85-88	218	13%	12-15	1624
Owner Income	<$20,000	638	86%	83-88	104	14%	12-17	742
	$20,000-39,999	2032	86%	84-87	339	14%	13-16	2371

	$40,000-59,999	3368	88%	87-89	464	12%	11-13	3832
	$60,000-79,999	3923	88%	87-89	523	12%	11-13	4446
	$80,000-99,999	3805	88%	87-89	510	12%	11-13	4315
	$100,000-119,999	3966	89%	88-90	496	11%	10-12	4462
	$120,000-139,999	2846	89%	88-90	350	11%	10-12	3196
	$140,000-159,999	2368	89%	88-91	281	11%	9-12	2649
	$160,000-179,999	1643	91%	90-92	164	9%	8-10	1807
	$180,000 or more	6798	90%	89-90	790	10%	10-11	7588
