# Supplementary material for: Supplement use is common in Dog Aging Project participants, especially among dogs with orthopedic conditions, and varies by life stage
Source: Am J Vet Res. Author manuscript; Available in PMC 2026 Jul 11. (PMC13355649; doi:10.2460/ajvr.25.06.0217)
Supplement: Supplementary Table S4 [file NIHMS2157768-supplement-Supplementary_Table_S4.pdf]

Supplementary Table S4: Dog demographic variables and less frequent than daily supplementation versus no supplementation as reported by owners as part of the Dog Aging Project initial survey, 2020-2022

Variable	Levels	No sup.	%	95% CI	Less than daily sup.	%	95% CI	Total
Breed Status	Mixed breed	17778	89%	89-90	2180	11%	10-11	19958
	Purebred	17967	88%	88-89	2404	12%	11-12	20371
Sex Class	Female, intact	1451	85%	84-87	250	15%	13-16	1701
	Female, spayed	16244	89%	88-89	2043	11%	11-12	18287
	Male, intact	2340	87%	85-88	365	13%	12-15	2705
	Male, neutered	15710	89%	89-90	1926	11%	10-11	17636
Lifestage	Puppy	1700	91%	90-93	162	9%	7-10	1862
	Young Adult	7371	89%	88-90	916	11%	10-12	8287
	Mature Adult	20071	89%	88-89	2606	11%	11-12	22677
	Senior	6566	88%	87-89	897	12%	11-13	7463
Dog Primary Purpose	Companion	34019	89%	88-89	4316	11%	11-12	38335
	Obedience	259	90%	86-93	30	10%	7-14	289
	Show	63	90%	80-96	7	10%	4-20	70
	Breeding	50	82%	70-91	11	18%	9-30	61
	Agility	99	88%	81-94	13	12%	6-19	112
	Hunting	50	89%	78-96	6	11%	4-22	56
	Working	98	80%	72-87	24	20%	13-28	122
	Service	325	84%	80-87	62	16%	13-20	387
	Search and Rescue	54	78%	67-87	15	22%	13-33	69
	Therapy or Service	234	84%	79-88	45	16%	12-21	279
Activity Level	Very Active	7199	89%	88-90	887	11%	10-12	8086
	Moderately Active	23946	88%	88-89	3130	12%	11-12	27076

	Not Active	4600	89%	88-90	567	11%	10-12	5167
General Health	Excellent	17761	90%	90-90	1988	10%	10-10	19749
	Very Good	12052	87%	87-88	1766	13%	12-13	13818
	Good	4347	88%	87-89	610	12%	11-13	4957
	Fair	1302	89%	87-90	168	11%	10-13	1470
	Poor	236	85%	80-89	42	15%	11-20	278
	Very Poor	47	82%	70-91	10	18%	9-30	57
Vet Visit Frequency	More than once per year	19490	88%	88-88	2654	12%	12-12	22144
	About once per year	15445	90%	89-90	1801	10%	10-11	17246
	Less than once per year	763	86%	84-88	122	14%	12-16	885
	Never	47	87%	75-95	7	13%	5-25	54
