# Supplementary material for: Supplement use is common in Dog Aging Project participants, especially among dogs with orthopedic conditions, and varies by life stage
Source: Am J Vet Res. Author manuscript; Available in PMC 2026 Jul 11. (PMC13355649; doi:10.2460/ajvr.25.06.0217)
Supplement: Supplementary Table S2 [file NIHMS2157768-supplement-Supplementary_Table_S2.pdf]

Supplementary Table S2: Dog demographic variables and daily supplementation as reported by owners as part of the Dog Aging Project initial survey, 2020-2022

Variable	Levels	No daily sup.	%	95% CI	Daily sup. use	%	95% CI	Total
Breed	Mixed breed	11606	58%	57-59	8352	42%	41-43	19958
	Purebred	10176	50%	49-51	10195	50%	49-51	20371
Sex	Female	10851	46%	45-47	9137	39%	38-39	23554
	Male	10931	46%	45-46	9410	39%	39-40	23890
Neuter Status	Intact	2326	42%	41-43	2080	37%	36-39	5556
	Neutered	19456	46%	46-47	16467	39%	39-40	41888
Life Stage	Puppy	1180	63%	61-66	682	37%	34-39	1862
	Young Adult	5348	65%	63-66	2939	35%	34-37	8287
	Mature Adult	12346	54%	54-55	10331	46%	45-46	22677
	Senior	2876	39%	37-40	4587	61%	60-63	7463
Dog primary purpose	Companion	20831	54%	54-55	17504	46%	45-46	38335
	Obedience	149	52%	46-57	140	48%	43-54	289
	Show	30	43%	31-55	40	57%	45-69	70
	Breeding	26	43%	30-56	35	57%	44-70	61
	Agility	35	31%	23-41	77	69%	59-77	112
	Hunting	33	59%	45-72	23	41%	28-55	56
	Working	65	53%	44-62	57	47%	38-56	122
	Service	172	44%	39-50	215	56%	50-61	387
	Search and Rescue	27	39%	28-52	42	61%	48-72	69
	Assistance or Therapy	137	49%	43-55	142	51%	45-57	279
Activity Level	Very Active	4579	57%	56-58	3507	43%	42-44	8086
	Moderately Active	14589	54%	53-54	12487	46%	46-47	27076
	Not Active	2614	51%	49-52	2553	49%	48-51	5167
General Health	Excellent	11871	60%	59-61	7878	40%	39-41	19749
	Very Good	7082	51%	50-52	6736	49%	48-50	13818
	Good	2119	43%	41-44	2838	57%	56-59	4957

	Fair	573	39%	36-42	897	61%	58-64	1470
	Poor	112	40%	34-46	166	60%	54-66	278
	Very Poor	25	44%	31-58	32	56%	42-69	57
Vet Visit Freq	More than once per year	10899	49%	49-50	11245	51%	50-51	22144
	About once per year	10292	60%	59-60	6954	40%	40-41	17246
	Less than once per year	553	62%	59-66	332	38%	34-41	885
	Never	38	70%	56-82	16	30%	18-44	54
