# Supplementary material for: Supplement use is common in Dog Aging Project participants, especially among dogs with orthopedic conditions, and varies by life stage
Source: Am J Vet Res. Author manuscript; Available in PMC 2026 Jul 11. (PMC13355649; doi:10.2460/ajvr.25.06.0217)
Supplement: Supplementary Table S1 [file NIHMS2157768-supplement-Supplementary_Table_S1.pdf]

Supplementary Table S1: Non-individual supplement codes applied to “other” free text responses in daily supplements received by Dog Aging Project dogs, 2020-2022.

Category code	Responses contained	e.g. and comments
Algae non-omega	Spirulina, chlorella or spelling variations of these two (e.g. chorella)	
Allergy	“allergy supplement” or similar phrasing	CBD allergy drops<br>Colostrum during allergy season<br>Named brand “allergy chews”
Anal gland	“Anal gland supplement” or similar phrasing	Named brand anal gland supplement (glandex)
Bladder	Bladder support or non-specific “urinary supplement”	
Brain	“brain supplement” or similar phrasing	Named brand (senilife)
Calming	Contained a named brand supplement intended for calming, or non-specific “calming supplement” text	Named brand (zylkene, composure, solliquin), L-theanine
Cardiac	Contained a named brand supplement intended for cardiac health	
Chinese herb	Contained “Chinese herb” or a specific name in the response (e.g. Yunnan baiyao)	
Coprophagia	Response contained “poop-eating deterrent”, “coprophagia prevention” or some variation thereof	
Dental	“dental” or “for teeth” mentioned	Named brand (plaque-off, ora-vet chews, greenies)
Eye	“for eyes”/ “eye support” / “vision support” or lutein mentioned – *different* from tear stain supplement code.	Named brand (optixcare, ocluvet, ocu-glo and alternate spellings thereof)
Fiber	Fiber, pumpkin, psyllium	
Food	A non-extract version of a whole food item was mentioned. This category did not include responses which were lists of ingredients from commercially prepared diets.	Chia seeds, goats milk, egg, etc.
Liver support	Contained milk thistle, SAM-e, “liver supplement”	Named brand (denamarin or alternate spellings thereof)

Muscle	Named brand supplement, or “muscle supplement” / “muscle enhancement”	Named brand (MyOs)
Mushroom	Included “mushroom” or named types of mushrooms (e.g. turkey tail, reishi, etc.).	This included food mushrooms, and “medicinal mushrooms”
Omega	Contained DHA, EPA, fish oil, krill oil, cod liver oil,	Named brand (welactin)
Other joint	Green lipped mussels (and alternate spellings thereof), MSM, collagen, named product which contains more than glucosamine/chondroitin hyaluronic acid, or non-specific “joint supplement”	Named brand (glycoflex, dasuquin)
Tear Stain	Named brand product or “to help with black eye discharge”/ “for tear stains”	Named brand (Angel Eyes, miracle eyes)
