# Supplementary material for: Supplement use is common in Dog Aging Project participants, especially among dogs with orthopedic conditions, and varies by life stage
Source: Am J Vet Res. Author manuscript; Available in PMC 2026 Jul 11. (PMC13355649; doi:10.2460/ajvr.25.06.0217)

Supplementary Figure S1: Percentage of dogs receiving any type of supplement at daily and less frequent than daily rates of administration as reported by owners enrolling in the Dog Aging Project, 2020-2022.

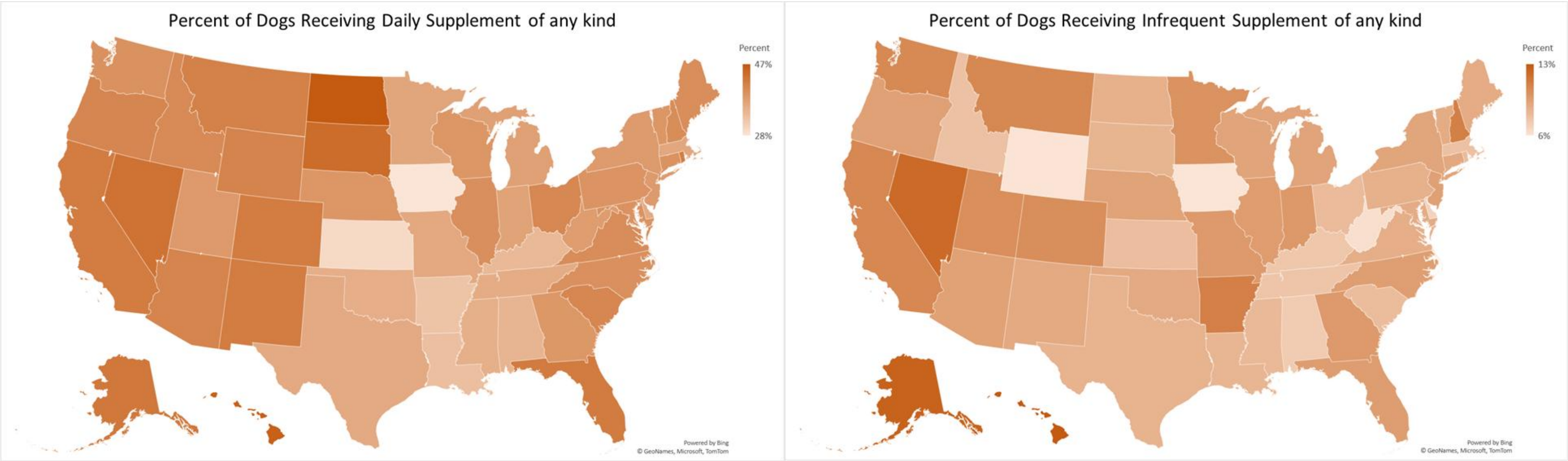

Supplement: Supplementary Figure S1 [file NIHMS2157768-supplement-Supplementary_Figure_S1.pdf]
